# Chronic Arsenic Exposure Upregulates the Expression of Basal Transcriptional Factors and Increases Invasiveness of the Non-Muscle Invasive Papillary Bladder Cancer Line RT4

**DOI:** 10.3390/ijms232012313

**Published:** 2022-10-14

**Authors:** Aaron A. Mehus, Nicholas Bergum, Peter Knutson, Swojani Shrestha, Matthew Kalonick, Xudong Zhou, Scott H. Garrett, Donald A. Sens, Mary Ann Sens, Seema Somji

**Affiliations:** Department of Pathology, School of Medicine and Health Sciences, University of North Dakota, Grand Forks, ND 58202, USA

**Keywords:** arsenite, heavy metal, urothelial carcinoma, luminal, basal

## Abstract

The bladder is a target organ for inorganic arsenic, a carcinogen and common environmental contaminant found in soil and water. Urothelial carcinoma (UC) is the most common type of bladder cancer (BC) that develops into papillary or non-papillary tumors. Papillary tumors are mostly non-muscle invasive (NMIUC), easier treated, and have a better prognosis. Urothelial carcinoma can be molecularly sub-typed as luminal or basal, with papillary tumors generally falling into the luminal category and basal tumors exclusively forming muscle invasive urothelial carcinomas (MIUC). It is unclear why some UCs develop more aggressive basal phenotypes. We hypothesized that chronic arsenic exposure of a papillary luminal bladder cancer would lead to the development of basal characteristics and increase in invasiveness. We treated the human papillary bladder cancer cell line RT4 with 1 µM arsenite (As^3+^) for twenty passages. Throughout the study, key luminal and basal gene/protein markers in the exposed cells were evaluated and at passage twenty, the cells were injected into athymic mice to evaluate tumor histology and measure protein markers using immunohistochemistry. Our data indicates that chronic As^3+^- treatment altered cellular morphology and decreased several luminal markers in cell culture. The histology of the tumors generated from the As^3+^-exposed cells was similar to the parent (non-treated) however, they appeared to be more invasive in the liver and displayed elevated levels of some basal markers. Our study demonstrates that chronic As^3+^ exposure is able to convert a non-invasive papillary bladder cancer to an invasive form that acquires some basal characteristics.

## 1. Introduction

Bladder cancer (BC) is one of the most common cancers in the world and it is estimated that approximately 81,400 new cases will be diagnosed in the US in the year 2020 [1]. The incidence of bladder cancer is higher in men with a male-to-female ratio of 3:1 [2]. BC develops mostly in the 6th to 7th decade of life [3,4] with the median age at diagnosis being 69 years for men and 71 years for women [5].

Among bladder cancers, the most common form is urothelial cancer (UC) that arises from the urothelial cells of the bladder mucosa [6,7] and can develop into papillary or non-papillary tumors. Papillary tumors are superficial and non-muscle invasive (NMIUC), whereas non-papillary tumors generally develop into muscle invasive forms (MIUC) [8]. Histologically there are 2 precursor lesions that lead to the development of muscle invasive urothelial carcinoma: the non-invasive papillary tumors and flat noninvasive urothelial carcinoma in situ (CIS). The non-invasive papillary tumors are common and frequently recur with approximately 20% of the cases progressing to the muscle invasive type. The majority of the MIUCs progress from CIS [8]. The NMIUCs as well as the MIUCs are sub-typed into various groups based upon the expression patterns of certain genes with the luminal and basal sub-type being the most prominent. The luminal sub-type includes the NMIUCs as well as the some of the MIUCs. This sub-type is less aggressive and includes tumors with a papillary histology with patients having a more favorable outcome. The basal sub-type tumors are more aggressive, and have a poor overall patient survival. They often exhibit squamous differentiation and are found exclusively in MIUC that metastasize to distal organs [9,10,11,12].

A number of environmental risk factors are implicated in the development of urothelial cancer with the foremost being cigarette smoking [2,5,13,14]. Occupational exposures to aromatic amines found in products from chemical, dye and rubber industries, hair dyes, paints plastics, and motor vehicle exhaust are also implicated in the development of bladder cancer [15,16,17,18,19,20,21].

Another risk factor associated with the development of bladder cancer is exposure to the metalloid arsenic, a natural element found in rocks, soil, and water, as well as agricultural and industrial sources [22]. Studies have shown that exposure to arsenic in the drinking water is associated with an increase risk to developing internal cancers such as bladder cancer in human populations in Taiwan [23,24,25], Chile [26] and Argentina [27,28]. In our previous studies, we developed an in vitro model of arsenic induced bladder cancer [29]. This was achieved by exposing a normal non-tumorigenic immortalized urothelial cell line, UROtsa to 1 µM arsenite (As^3+^) for an extended period of time. The transformed cell lines formed tumors in athymic mice and displayed features similar to urothelial carcinomas with focal areas of squamous differentiation [29,30], an indicator of poor prognosis [31,32]. Immunohistochemical analysis of the tumors showed an increase in expression of keratins (KRT) 1, 5, 6, 14 and 16, which are associated with the basal subtype of urothelial carcinoma [33].

Urothelial cancers take a long time to develop and currently it is not known if long-term exposure to arsenite (As^3+^) could convert a papillary non-muscle invasive urothelial cancer to a more aggressive form that has the potential to metastasize and spread to distal organs. A recent study found that long-term exposure of the breast cancer cell line MCF-7 (luminal A sub-type) to As^3+^ converts it to a more aggressive metastatic form with expression of basal markers [34]. This study supports the notion that As^3+^ might play a similar role for the progression of papillary NMIUC luminal urothelial cancer to a more basal genotype and phenotype. In breast cancer patient studies, As^3+^ does not seem to have a strong association with the development and progression of the disease however, as mentioned above; it does have a strong association with the development and progression of urothelial cancer. This renders As^3+^ as a viable candidate to test for its ability to advance a papillary NMIUC to MIUC. Thus, to determine if a papillary urothelial cancer can become basal in character and invade surrounding tissue, a suitable urothelial cancer cell line is required. Currently there are 103 bladder cancer cell lines that have been cited in research publications and 69 have been profiled by at least one “omic” technology [35]. However, only eight bladder cancer cell lines have been submitted, characterized, and accepted by the American Type Culture Collection (ATCC, https://www.atcc.org/, accessed on 1 October 2021). Of these eight cell lines, RT4 is the only one that has characteristics consistent with a luminal, papillary non-muscle invasive urothelial carcinoma [36]. In this study, we sought to determine if long-term exposure to As^3+^ could affect the molecular characteristics of a papillary luminal UC. In addition, we also wanted to determine if As^3+^ exposure could convert a papillary NMIUC into a muscle invasive form that could metastasize into the tissue structures.

## 2. Results

### 2.1. Morphology and Growth Rates of As^3+^-Exposed RT4 Cells

**Figure 1:**
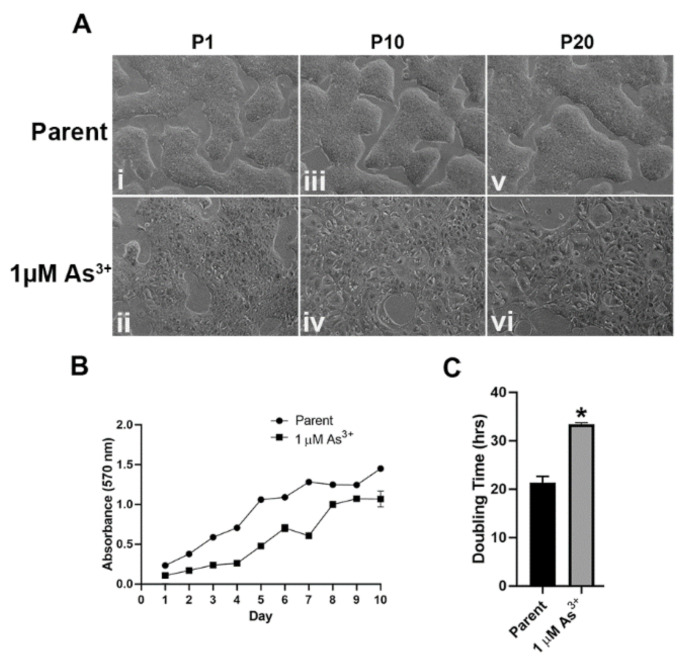
Cellular morphology and growth rates of As^3+^-exposed RT4 cells. (**A**) The cellular morphology of the parent (non-treated (**i**,**iii**,**v**)) and 1 µM As^3+^-exposed cells (**ii**,**iv**,**vi**) was monitored at passages 1, 10, and 20 (P1, P10, and P20). All images are at 100× magnification. (**B**) The cellular growth of the parent (circles) and 1 µM As^3+^ (squares) was assessed using methylthiazoletetrazolium (MTT) assay following a 1:20 subculture of the cells at P20. (**C**) The calculated doubling time of the cell lines presented in hours at P20. The measurements were performed in triplicates and the values reported are mean percentage of control ± SEM. An unpaired *t*-test was performed. Asterisks indicate significant differences from the passaged-matched parent (*p* < 0.05).

RT4 cells were cultured in the presence of 1 µM As^3+^ for 20 passages. There was a change in the morphology of the cells after one passage (Figure 1A(i,ii)) with cells loosely packed, and this change became more pronounced after 10 and 20 passages where the cells spread out and were more flattened, larger in size, and no longer tightly connected to each other (Figure 1A(iii–vi)) when compared to the passage matched unexposed cells. The growth rates of the As^3+^ exposed cells decreased significantly after 20 passages (Figure 1B) with the unexposed cells demonstrating a doubling time of 21.39 ± 1.29 h and the 1 µM As^3+^ exposed cells demonstrating a doubling time of 33.40 ± 0.36 h (Figure 1C). Thus, long-term exposure to As^3+^ altered the morphology as well as the growth rate of RT4 cells.

### 2.2. Expression of Luminal Genes and Proteins in As^3+^ Exposed RT4 Cells

**Figure 2:**
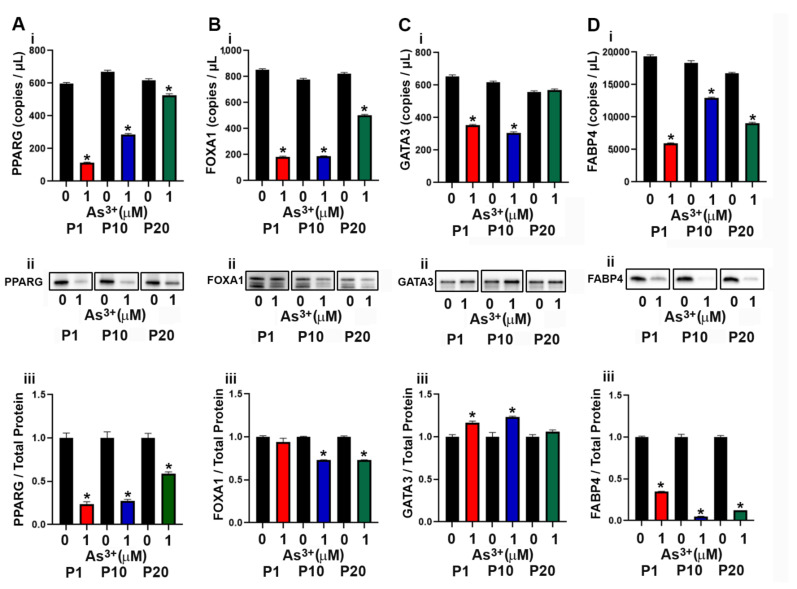
Expression of luminal markers in parent and As^3+^-exposed RT4 cells. (**Ai**–**Aiii**) PPARγ expression, (**Bi**–**Biii**) FOXA1 expression, (**Ci**–**Ciii**) GATA3 expression, and (**Di**–**Diii**) FABP4 expression in parent and 1 µM As^3+^-exposed RT4 cells at passages 1, 10, and 20 (P1, P10, and P20). Images are cropped to show relevant bands. Uncropped Western blots are shown in Supplementary Figure S2. Droplet digital PCR (ddPCR) analysis was performed to verify gene expression (**Ai**–**Di**). Western blot analysis was used to measure protein levels (**Aii**–**Dii**) and the integrated optical density (IOD) of each protein band was calculated (**Aiii**–**Diii**). Gene expression is reported as copies per µL and protein expression was normalized to total protein per lane and then plotted as fold-change compared to the parent. Triplicate measurements of gene and protein data were performed and are reported as mean ± SEM. An unpaired *t*-test was performed. Asterisks indicate significant differences from the passaged-matched parent (*p* < 0.05).

The development of the luminal subtype of urothelial cancer depends upon the expression of the transcriptional factors PPARγ, FOXA1 and GATA3 [37,38]. In a previous study, exposure to As^3+^ resulted in a decrease in expression of PPARγ in mesenchymal stem cells resulting in the inhibition of adipogenic differentiation [39]. In this study the effect of continuous As^3+^ exposure for 20 passages on the expression levels of PPARγ and other luminal transcriptional factors in the RT4 (luminal) cell line was determined. As seen in Figure 2A(i–iii), the expression of PPARγ decreased at P1 with 1 µM As^3+^ exposure and remained low up to 10 passages. At passage 20, the levels of PPARγ increased but were still lower than that of the control cells. The gene and protein expression levels of FOXA1 (Figure 2B(i–iii)) was decreased with As^3+^ exposure at P10 and P20 compared to control cells, however, FOXA1 protein at P1 was not reduced despite attenuated gene expression. The mRNA levels for GATA3 were low in the 1 µM As^3+^-exposed cells compared to the parent at P1 and P10 but returned to parent levels at P20 (Figure 2Ci). The protein levels for As^3+^-exposed cells were elevated at P1 and P10 but returned to the level of the parent at P20 (Figure 2C(ii–iii)). The expression of FABP4, a PPARγ regulated protein in urothelial cancer [40] was also determined and exposure to As^3+^ decreased the expression at all 3 passages (Figure 2D(i–iii)). We also determined the expression of KRT13, a KRT expressed in the intermediate layers of the urothelium [41]. These intermediate cells give rise to the luminal subtype of urothelial carcinoma and our results showed that expression of KRT13 mRNA in the RT4 cell line was low and treatment with As^3+^ further decreased the expression of this gene (Supplemental Figure S1A). There was no expression of the protein in the RT4 control and the As^3+^-exposed cell lines.

### 2.3. Expression of Basal Genes and Proteins in As^3+^ Exposed RT4 Cells

**Figure 3:**
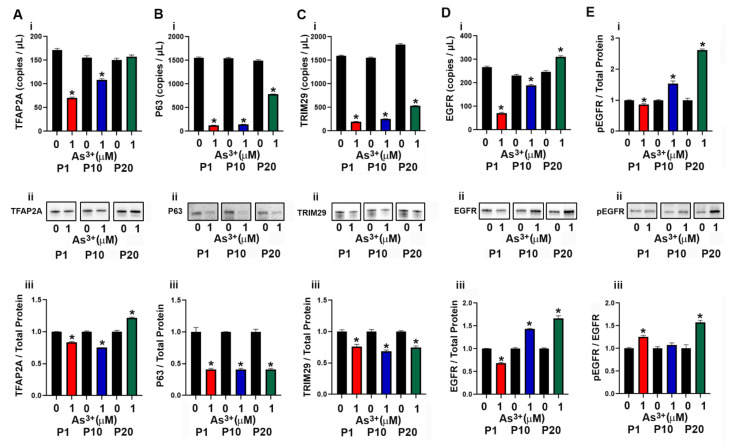
Expression of basal markers in parent and As^3+^-exposed RT4 cells. (**Ai**–**Aiii**) TFAP2A expression, (**Bi**–**Biii**) P63 expression, (**Ci**–**Ciii**) TRIM29 expression, (**Di**–**Diii**) EGFR expression, and (**Ei**–**Eiii**) phosphorylation of EGFR in parent and 1 µM As^3+^-exposed RT4 cells at passages 1, 10, and 20 (P1, P10, and P20). Images are cropped to show relevant bands. Uncropped Western blots are shown in Supplementary Figure S2. Droplet digital PCR (ddPCR) analysis was performed to verify gene expression (**Ai**–**Di**). Western blot analysis was used to measure protein levels (**Aii**–**Eii**) and the integrated optical density (IOD) of each protein band was calculated (**Aiii**–**Eiii**). Ratio of pEGFR over total EGFR expression in parent and 1 μM As^3+^ RT4 cells. Gene expression is reported as copies per µL and protein expression was normalized to total protein per lane and then plotted as fold-change compared to the parent. Triplicate measurements of gene and protein data were performed and are reported as mean ± SEM. An unpaired *t*-test was performed. Asterisks indicate significant differences from the passaged-matched parent (*p* < 0.05).

The basal subtype of urothelial cancer is characterized by the expression of high molecular weight keratins (KRT5, KRT6 and KRT14) [9] as well as the transcriptional factors P63 [42] and TFAP2A [43]. The expression of KRT5 in the As^3+^-exposed cells was very low and the mRNA levels were undetected at several of the time points (Supplemental Figure S1B). There was no KRT5 protein detected at any of the passages. The expression levels of KRT6 and KRT14 were also undetected at any of the passages. The transcription factor TFAP2A is expressed in basal/squamous urothelial cancers and it facilitates the expression of the transcription factor P63 [42,43] which is associated with an increase in expression of TRIM29 and KRT14 [44]. In our study, exposure to As^3+^ at P1 and P10 decreased the expression of TFAP2A, whereas at P20, the protein expression level increased (Figure 3A(i–iii)). The expression levels of P63 and TRIM29 decreased with As^3+^ exposure at all passages (Figure 3B(i–iii),C(i–iii)). Previous studies have shown that the expression of epidermal growth factor receptor (EGFR) is also associated with the basal subtype of bladder cancer [37] and in our study, exposure to As^3+^ increased the expression as well as the phosphorylation of the receptor (pEGFR, Figure 3D(i–iii),E(i–iii)) at passages 10 and 20.

### 2.4. Effect of As^3+^ Exposure on the Expression of Epithelial to Mesenchymal Transition (EMT) Genes and Proteins in RT4 Cells

**Figure 4:**
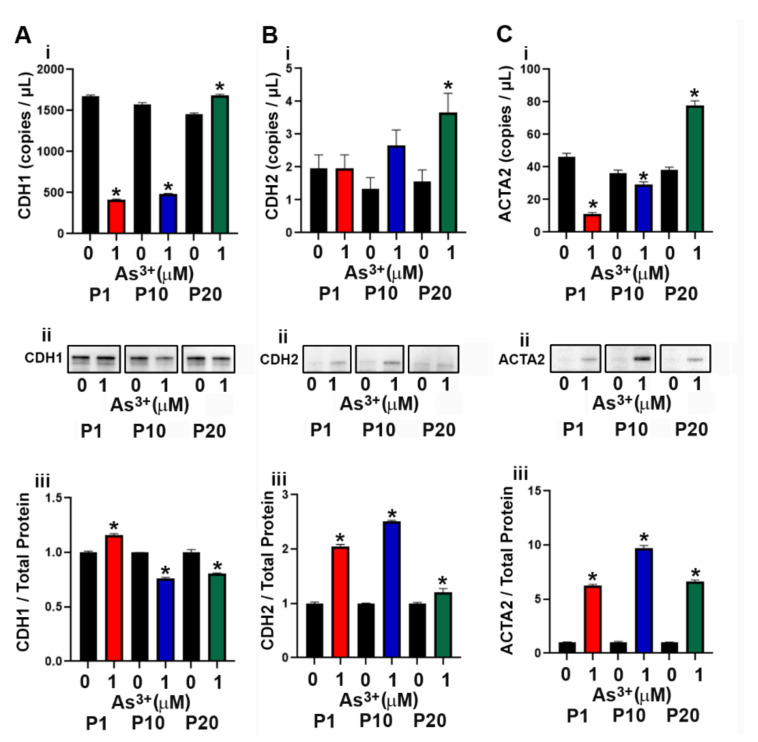
Expression of epithelial to mesenchymal transition (EMT) markers in RT4 cells exposed to As^3+^. (**Ai**–**Aiii**) CDH1 expression, (**Bi**–**Biii**) CDH2 expression, and (**Ci**–**Ciii**) ACTA2 expression in parent and 1 µM As^3+^-exposed RT4 cells at passages 1, 10, and 20 (P1, P10, and P20). Images are cropped to show relevant bands. Uncropped Western blots are shown in Supplementary Figure S2. Droplet digital PCR (ddPCR) analysis was performed to verify gene expression (**Ai**–**Ci**). Western blot analysis was used to measure protein levels (**Aii**–**Cii**) and the integrated optical density (IOD) of each protein band was calculated (**Aiii**–**Ciii**). Gene expression is reported as copies per µL and protein expression was normalized to total protein per lane and then plotted as fold-change compared to the parent. Triplicate measurements of gene and protein data were performed and are reported as mean ± SEM. An unpaired t-test was performed. Asterisks indicate significant differences from the passaged-matched parent (*p* < 0.05).

The RT4 cell line was isolated from a T2 papillary cell carcinoma with superficial invasion of the bladder muscle wall [45] and the effect of As^3+^ exposure on the expression of genes involved in the EMT process was determined. As seen in Supplemental Figure S1C–E, the expression of SNAI1 and SNAI2 mRNA did not increase with As^3+^ exposure, whereas the expression of TWIST1 was increased at P10 and P20 in the exposed cells. The protein levels for SNAI1, SNAI2 and TWIST1 were not detected by Western analysis. The mRNA levels for CDH1 (E-cadherin) decreased at P1 and P10 with the protein levels initially increasing at P1 but decreased at P10 and P20 (Figure 4A(i–iii)). The protein expression of CDH2 (N-cadherin) increased with As^3+^ exposure in all passages (Figure 4B(i–iii)). The protein expression of ACTA2 (smooth muscle alpha (α)-2 actin) was elevated at all passages with 1 µM As^3+^ exposure while the mRNA was only elevated at P20 (Figure 4C(i–iii)).

### 2.5. Histology of the As^3+^-Exposed Tumor Heterotransplants

**Figure 5:**
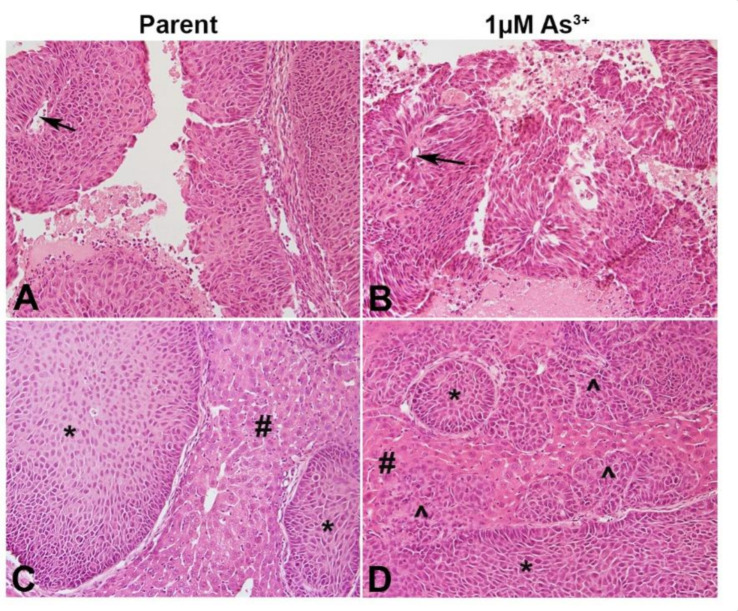
Histology of the tumors produced by the RT4 cell lines. (**A**) SQ tumor formed by RT4 parent cells. Non-invasive papillary carcinoma is visible with distinct papillary core (arrow). (**B**) SQ tumor formed by As^3+^-treated RT4 cells. Non-invasive papillary carcinoma is visible with distinct papillary core (arrow). (**C**) IP tumor formed by RT4 parent cells. Focal tumor nests (*) seen in the liver (#). (**D**) IP tumor formed by 1 µM As^3+^-treated RT4 cells. Focal tumor nest (*) as well as invasive carcinoma (^) seen in the liver (#). Invasive sheets of cancer seen in the liver as indicated by the arrows. All images are at a magnification of 200×.

The ability of the RT4 cell to for papillary tumors when injected SQ was determined. As seen in Figure 5A,B, both the non-treated as well as the As^3+^-treated cells formed papillary tumors with a well-defined visible core. Since urothelial cancers tend to spread locally after escaping from the bladder and they colonize organs particularly the liver within the peritoneal cavity, the ability of the As^3+^-exposed RT4 cells to form tumors in the liver was determined. Histologically, the parent RT4 cells showed focal involvement of the liver with tumor nests (Figure 5C), while the As^3+^-exposed cells showed prominent liver involvement with sheets and cords of invasive carcinoma (Figure 5D). There was no evidence of keratinization and intercellular bridging with H&E staining.

### 2.6. Immunohistochemical Analysis of As^3+^-Exposed Tumor Heterotransplants

**Figure 6:**
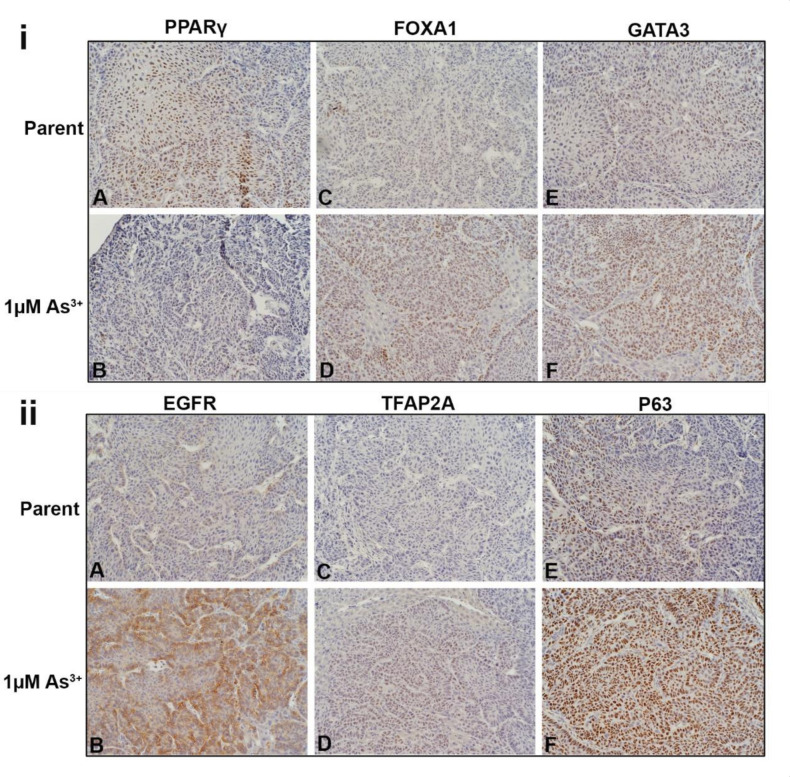

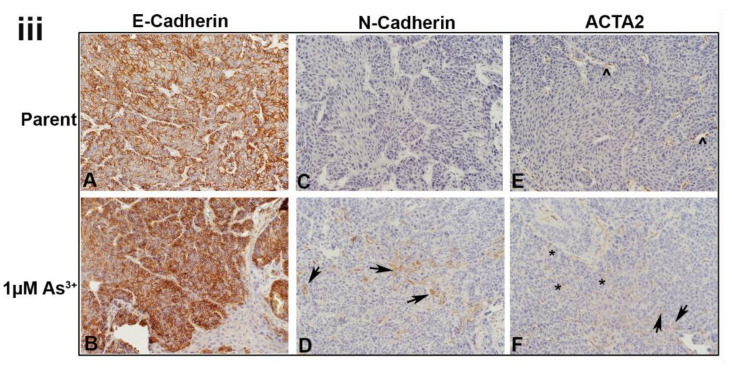
Immunohistochemical staining of luminal, basal and EMT markers within the tumors formed from parent and As^3+^-exposed RT4 cells. (**iA**,**iB**). Nuclear PPARγ expression in the tumors produced by the parent and As^3+^-exposed cells, PPARγ was expressed in the majority of cells in the parent and weaker in the 1 µM As^3+^-treated cells. (**iC**,i**D**). Nuclear FOXA1 expression, there was a greater proportion of staining of FOXA1 in the tumors generated from the 1 µM As^3+^-exposed cells compared to the parent. (**iE**,i**F**). Nuclear expression of GATA3, having greater staining within the tumors generated from cells exposed to 1 µM As^3+^ compared to the tumors formed by the parent RT4 cells. (**iiA**,**iiB**). Membrane staining of EGFR in tumors produced by the parent and As^3+^-treated cells. EGFR expression was higher in the tumors produced from 1 µM As^3+^ treatment compared to the parent. (**iiC**,**iiD**). Nuclear staining of TFAP2A, having greater expression in tumors produced from 1 µM As^3+^ treatment compared to the parent. (**iiE**,**iiF**). Nuclear expression of P63, showing greater staining in tumors produced from 1 µM As^3+^ treatment compared to the parent. (**iiiA**,**iiiB**). Membrane expression of E-Cadherin (CDH1) in tumors produced by the parent (**iiiA**) and As^3+^-treated cells (**iiiB**), the staining was strong in all the tumors. (**iiiC**,**iiiD**). Membrane staining of N-Cadherin (CDH2), the parent displayed no expression but there was focal staining in the As^3+^-exposed tumors with a few cells expressing the protein localized to the cell membrane (black arrows). (**iiiE**,**iiiF**). Staining for ACTA2, in the parent expression was mainly in the cells of the blood vessels and connective tissue (**iiiE**). In the tumors formed by the As^3+^-exposed cells (**iiiF**), there were more blood vessels which stained for ACTA2 and there was also an increase in staining in the connective tissue cells. A few dispersed tumor cells also showed staining for the protein (**iiiF**). The black arrows point to tumor cells, the caret (^) indicates blood vessels, and the asterisks (*) point out the stroma/connective tissue. The brown color indicates the presence of the protein whereas the blue/purple color indicates the nuclei that are stained with the counterstain hematoxylin. All images are at a magnification of 200×.

The liver tumor transplants were stained for luminal, basal and EMT proteins. The intensity and the approximate percent of tumor staining for each marker is shown in Table 1. Figure 6i shows the nuclear expression of PPARγ, FOXA1, and GATA3 in the tumors. In the tumors produced by the parent cells (Figure 6iA), the expression of PPARγ was strong with approximately 70% of the cells expressing the protein. The expression of PPARγ was weaker in the tumors produced by the 1 µM As^3+^-exposed cells (Figure 6iB) with approximately 10% of the tumor cells expressing the protein. There was a greater proportion of cells (approximately 60%) staining for FOXA1 in the tumors generated from the 1 µM As^3+^-exposed cells (Figure 6iD) compared to the parent (Figure 6iC). The staining for GATA3 was moderate within the tumors generated from cells exposed to 1 µM As^3+^ (Figure 6iF) compared to the tumors formed by the parent (Figure 6iE). There was no staining for FABP4 in any of the tumors. The cytoplasmic expression of KRT13 was strong in the tumors formed by the parent RT4 cells with most of the cells staining for the protein (Supplemental Figure S3A), whereas in the tumors formed by the As^3+^-exposed cells, the staining for KRT13 was focal with moderate staining (Supplemental Figure S3B).

The cytoplasmic staining pattern for KRT5 was similar in the parent and the As^3+^-exposed RT4 tumors (Supplemental Figure S3C,D), however, the intensity appeared to be lower in the 1 µM As^3+^-exposed cells (Supplemental Figure S3D). There was no expression of KRT6, KRT14, KRT16, or TRIM29 in the RT4 tumors. The membrane staining for EGFR in the tumors generated from the parent RT4 cells was weak (Figure 6iiA) with staining in approximately 10 % of the cells, whereas the tumors formed by the As^3+^-exposed cells (Figure 6iiB) had a majority of cells (approximately 80%) stain moderately for the receptor. There was no staining for the pEGFR in any of the tumors. There was no staining for TFAP2A in the tumors formed by the parent cells (Figure 6iiC), whereas the tumors formed by the 1 µM As^3+^-exposed cells (Figure 6iiD) displayed weak nuclear staining for the protein in approximately 60% of the cells. The staining for P63 in the parent RT4 tumor was moderate with approximately 30% of the tumor showing nuclear staining (Figure 6iiE) whereas the tumors formed by the As^3+^-exposed cells had a majority of the cells (80%) showing strong nuclear staining (Figure 6iiF).

All the tumors showed strong membrane staining for E-cadherin in the majority of the cells (Figure 6iiiA,B). There was no staining of N-cadherin in the parent RT4 tumors (Figure 6iiiC), however, there was focal staining in the As^3+^-exposed tumors with a few cells (10%) expressing the protein localized to the cell membrane (Figure 6iiiD). The staining for ACTA2 in the parent RT4 tumors was mainly in the cells of the blood vessels and connective tissue (Figure 6iiiE). In the tumors formed by the As^3+^-exposed cells, there were more blood vessels which stained for ACTA2 and there was also an increase in staining in the connective tissue cells. A few dispersed tumor cells also showed staining for the protein (Figure 6iiiF).

## 3. Discussion

The role of carcinogens in the progression of bladder cancers from a less aggressive state to a more aggressive one is not well established. In the present study, the effect of long-term arsenic exposure on the molecular signature and tumorigenicity of the urothelial cancer cell line RT4 was determined. This cell line was isolated from a high grade papillary urothelial cancer [46] and when injected in immune-compromised mice formed non-invasive papillary tumors similar to human papillary urothelial cancers [43]. Molecular analysis of this cell line demonstrated a luminal molecular pattern [12,43]. In our study, the RT4 cells were cultured in the presence of 1 µM As^3+^ (an environmentally relevant dose) and the changes in the gene and protein expression within the cell lines at the 1st, 10th and 20 passage was determined. The in vitro data demonstrates that long-term As^3+^ exposure decreases the cellular growth rate and abolishes the morphology seen in the parent (non-treated) RT4 cells. However, the in vivo data shows that there is no histological difference in the morphology of the cells in the tumors formed by the control and the As^3+^-exposed cells, although, invasion of the tumors into the liver was prominent in the As^3+^-exposed cells compared to the control cells, which show focal involvement. Generally, metastasis of tumors occur with a loss of epithelial proteins (E-cadherin), loss of contacts between the cells, and the re-expression of mesenchymal proteins (N-cadherin). The repression of E-cadherin expression in urothelial cancers is induced by a number of transcription factors, which include SNAI1, SNAI2 and TWIST1 [47,48,49]. In our study, there was no change in the expression levels of SNAI1 and SNAI2 with As^3+^ exposure, however, the mRNA levels of TWIST1 increased with exposure. None of these transcriptional factors were detected in the tumors which is not unusual since the expression levels of these protein tends to be very low and they may not be expressed at all stages of tumor development and growth. The levels of E-cadherin decreased with As^3+^ exposure, with an increase in the levels of N-cadherin in cell culture. In the tumors, there was no change in the expression of E-cadherin. There was focal expression of N-cadherin in a few tumor cells indicating that that they had undergone EMT and had acquired a mesenchymal characteristic feature. In addition to EMT, invasion and metastasis of cancers including urothelial cancer require an interaction between the tumor cells and the cancer associated fibroblasts that express ACTA2 [49,50]. Tumor cells also express ACTA2 and in our study, exposure to As^3+^ resulted in an increase in expression of ACTA2 in some of the tumor cells as well as an increase in expression of ACTA2 in the fibroblasts associated with the tumor stroma. Therefore, our data suggests that exposure to As^3+^ can convert a low-grade papillary tumor to a more invasive type.

PPARγ, a nuclear receptor activates transcription of genes involved in terminal differentiation of adipocytes [51]. It is also expressed in the urinary bladder [52] and in luminal subtypes of urothelial cancers [12,43]. In normal urothelial cells, PPARγ activation induces a switch in the expression of keratins thus effecting the differentiation state of the cells [41]. In urothelial cancers, studies show that PPARγ plays a role in the development as well as progression of the disease by regulating the processes of cell proliferation, apoptosis, production of reactive oxygen species, and lipid metabolism [53,54,55,56]. In our previous study [57], as well as studies done by others [37], PPARγ activation promoted the differentiation of the basal subtype of urothelial cancer to a luminal subtype and this involved the cooperation of PPARγ with FOXA1 and GATA3. Both FOXA1 and GATA3 also play a role in the differentiation of urothelial cells in human bladder cancers, and the expression of PPARγ, FOXA1 and GATA3 is negatively correlated to the grade of the tumor [58,59,60,61]. In mesenchymal stem cells, As^3+^ exposure inhibits adipogenic differentiation by reducing the expression of PPARγ [62]. In our study, exposure to 1 µM As^3+^ decreased the expression of PPARγ and since PPARγ plays a role in urothelial differentiation, a decrease in its expression could potentially increase the growth of the tumor cells. We did not see an increase rate in tumor growth, but we found the tumors formed by the As^3+^-exposed cells to be more aggressive with prominent liver involvement forming cords and sheets of invasive carcinoma. Contrary to a decrease in the expression of PPARγ in the 1 µM As^3+^ exposed tumors, the expression of FOXA1 and GATA3 increased. The reason for this increase is not known at present and it could be due to other factors that regulate the expression of these genes.

The expression of KRTs in the bladder is associated with the stage of differentiation with basal cells expressing both KRT5 and KRT14 and intermediate cells expressing only KRT5. The expression of KRT20 is restricted to the superficial umbrella layer of the bladder whereas the expression of KRT13 is restricted to the intermediate layer of the bladder [63]. These stages of differentiation also occur in bladder cancers with KRT14 expressed in the least differentiated bladder cancer and KRT20 expressed in the highly differentiated bladder cancers. In our previous study, we showed that As^3+^-transformed urothelial cells displayed a KRT profile similar to the basal subtype of urothelial cancer. The tumors formed by these transformed cells resembled urothelial cancers with areas of squamous differentiation, which is associated with poor prognosis. The squamous differentiated areas of the tumors stained for KRT5, 6 and 16, whereas the less differentiated areas resembling urothelial cancer stained for KRT5 and 14 [33]. In addition to expressing a unique KRT profile, the basal cancers with squamous differentiation also express the transcription factor P63 and TFAP2A [43]. TFAP2A can regulate the expression of P63, but it is not a driver of squamous differentiation [43] suggesting that other factors work in concert with TFAP2A to drive the process of squamous differentiation. The transcription factor P63 regulates the expression of the oncogene TRIM29 and KRT14 and both TRIM29 and KRT14 can promote the invasiveness of urothelial cancers [44]. Furthermore, in the TCGA bladder cancer data set, an increase correlation of TRIM29 was found with the basal genes P63, KRT5 and KRT6A suggesting a prominent role of these molecules in aggressive bladder cancers [44]. We have previously shown that normal urothelial cells transformed by As^3+^ form tumors which show strong staining for TRIM29, TFAP2A and P63 in the undifferentiated part of the tumor and weak staining in the areas of squamous differentiation [57]. In our current study, the tumors formed by the RT4 cells exposed to As^3+^ expressed elevated levels of basal transcriptional factors P63 and TFAP2A suggesting an active basal transcriptional machinery. However, this did not translate to an increase in the expression of any of the basal KRTs. A study performed by Fishwick et al. [38] demonstrated that siRNA knockdown of P63 in normal urothelial cells increased the expression of KRT13 suggesting a switch from the basal to a transitional epithelial differentiation program. In our study, treatment with As^3+^ resulted in an increase in expression of P63 in the tumors and a decrease in expression of KRT13. These changes were not seen in vitro suggesting that an in vivo environment is probably required to initiate these changes. It is possible that over time, the tumors would acquire a more basal character but due to the clinical condition of the animals, we were unable to extend the experiment.

Signaling via the EGFR is linked to the development and progression of urothelial cancers and an increase in the expression of EGFR is associated with the basal subtype of UC [64]. Many of the urothelial cancer cell lines sub-typed as basal show sensitivity to EGFR targeted therapy with inhibition of tumor growth in animal models [65]. Previous studies performed by us and others [37,41,57] showed that inhibition of cell proliferation via the EGFR pathway and activation of PPARγ with an agonist that promoted cell differentiation could convert a basal urothelial cell to a luminal type. In the present study, there was an increase in expression of EGFR along with a decrease in expression of PPARγ in tumors formed by the As^3+^-exposed RT4 cells, suggesting the activation of the basal differentiation program and a repression of the luminal differentiation program.

A majority of diagnosed human urothelial carcinomas are low-grade papillary NMIUC that generally fall into the luminal subtype. There is a fundamental lack in knowledge about the conversion of a luminal sub-type of urothelial cancer to a more aggressive basal sub-type. In our study we utilized the RT4 cell line which is the only commercially available cell line that represents a well-differentiated luminal, papillary NMIUC [36]. Future studies may involve utilizing commercial cell lines of the mixed or non-subtype of urothelial carcinoma. These cell lines display a low expression of both luminal and basal markers. Perhaps these non-subtype cell lines may be more susceptible to being converted from one subtype to another, but further work is needed to address that possibility.

In conclusion, ours is the first study to demonstrate that long-term exposure to As^3+^ converts a high-grade non-invasive papillary luminal subtype of bladder cancer to an invasive form that shows some of the changes associated with the basal subtype of urothelial cancer. This is an important finding since a small percent of papillary NMIUC’s progress to the muscle invasive form and the molecular mechanisms associated with the progression are unknown. Our study demonstrates that at least in an animal model, the progression involves the upregulation of some of the transcriptional factors associated with the basal subtype and the downregulation of some of the genes associated with the luminal subtype of urothelial cancer. Urothelial cancers develop slowly and it is possible that over time the tumors may acquire a more basal nature with squamous differentiation.

## 4. Materials and Methods

### 4.1. Animals

This study adhered to all recommendations dictated in the Guide for the Care and Use of Laboratory Animals of the NIH. The protocol was approved by The University of North Dakota Animal care Committee (IACUC# 1911-1C). Athymic nude (NCR-nu/nu) female mice were used in these studies. The mice were housed five to a cage at 22 °C under a 12 h light/dark cycle. Food and water was available ad libitum. Standard rodent diet (Envigo-8640, Teklad 22/5) was used, and selenium content was not altered between the groups. To assess the ability of the parent and As^3+^-treated RT4 cells to colonize internal organs of the peritoneum, the cells were injected subcutaneously (SQ) and intraperitoneally (IP) into 5 nude (NCr-nu/nu) mice per group. The SQ and IP injection of transformed cells has been described previously in detail [30,66]. The tumor size was assessed weekly using a ruler and the animals were sacrificed when the size of the tumor was approximately 1.5–1.8 cm or when dictated by clinical conditions. The animals were euthanized by CO_2_ asphyxiation and conformed to American Veterinary Medical Association Guideline on Euthanasia. Death was confirmed by ascertaining cardiac and respiratory arrest following which the tumors were harvested. Care was taken to ensure that there was no distress to the animals during the procedure.

### 4.2. Cell Culture

RT4 cells were purchased from ATCC and the STR authentication was provided from the company. The cells were tested for mycoplasma contamination prior to use in experimental protocol. The cells were maintained in 75-cm^2^ tissue culture flasks in Dulbeco’s modified Eagle’s medium (DMEM) supplemented with 10% *v*/*v* fetal bovine serum. The selenium content in the media was not altered and both As^3+^ and non-exposed cells received the same selenium concentrations from FBS. The confluent cells were sub-cultured at a 1:4 ratio using Trypsin-EDTA and the cultures were fed fresh growth medium every three days. Cultures were incubated at 37 °C in a 5% CO_2_:95% air atmosphere. For As^3+^ exposure, the cells were sub-cultured and maintained continuously in medium containing 1 μM NaAsO_2_ (Fluka Chemie AG, Cat# 71287, Buchs, Switzerland) as described previously [30] for 20 passages.

### 4.3. Cell growth

Growth curves of the parent and As^3+^-treated RT4 cells was assessed using the methylthiazoletetrazolium (MTT, 3-(4,5-dimethylthiazol-2-yl)-2,5-diphenyltetrazolium bromide) assay following a 1:20 subculture of the cells [67].

### 4.4. RNA Isolation and Droplet Digital PCR Analysis

Total RNA from cell pellets was isolated using Tri Reagent (Molecular Research Center, Cincinnati, OH, USA) as described previously [68]. The expression of various genes was assessed with droplet digital polymerase chain reaction (ddPCR) using primers that were purchased commercially from Bio-Rad Laboratories. The genes along with the catalog number of the primers are listed in Table S1. Total RNA (0.1 µg) was transcribed to cDNA using the iScript cDNA synthesis kit (Bio-Rad Laboratories, Hercules, CA, USA). Droplet digital PCR was performed with the QX200 Droplet Digital PCR system (Bio-Rad, Hercules, CA, USA). The ddPCR reaction mixture consisted of 12.5 μL of 2X EvaGreen master mix (Bio-Rad, Hercules, CA, USA), 1.25 µL Bio-Rad primers, 8.75 µL PCR H_2_O, and 2.5 µL cDNA in a final volume of 25 μL. Twenty microliters of each reaction mix was converted to droplets with the QX200 droplet generator (Bio-Rad, Hercules, CA, USA). After processing, the droplets generated from each sample were transferred to a 96-well PCR plate (Bio-Rad, Hercules, CA, USA). The plate containing droplets was then hot-sealed and PCR amplification was carried out on a T100 thermal cycler (Bio-Rad, Hercules, CA, USA) using a cycling protocol of 95 °C (enzyme activation) for 5 minutes followed by 40 cycles of a two-step cycling protocol of 95 °C for 30 seconds (denaturation) and 60 °C for 1 minute (annealing). Post-cycling parameters were 4 °C for 5 min, then enzyme deactivation at 90 °C for 5 min, followed by an infinite hold at 12 °C. The ramp rate between these steps was 2 °C/s; acquired data were analyzed with QuantaSoft Analysis Pro software (Bio-Rad, Hercules, CA, USA). Droplet digital PCR yields ~20,000 droplets/20 µL sample where each droplet is an independent PCR reaction of equal and defined volume. Therefore, ddPCR technology increases the signal-to-noise ratio while providing precise absolute quantitation without the need for a standard curve or reference [69]. The final data is reported as copies/µL.

### 4.5. Western Blot Analysis

Western blot analysis was performed as described previously [57]. The cell pellets were dissolved in 1X Radio-immunoassay Precipitation Assay (RIPA) lysis buffer supplemented with PMSF, protease inhibitor cocktail, and sodium orthovandate (Santa Cruz Biotechnology, Dallas, TX, USA). The cell suspension was sonicated, and the lysate was centrifuged to remove cellular debris. Protein lysates were quantified using the Pierce BCA Protein Assay Kit (Thermo-Scientific Pierce, Waltham, MA, USA). Prior to loading, samples were reduced and denatured. The protein extracts were separated on TGX AnykD SDS-polyacrylamide gels purchased from Bio-Rad laboratories and transferred to a 0.2 µm hybond-P polyvinylidene difluoride (PVDF) membrane using semi-dry transfer. Protein loading was normalized using PierceReversible Protein Stain Kit (Thermo Scientific) for PVDF Membranes which has been described elsewhere [70] and the resulting signal of each lane was quantified using Image Lab software (version 4.1, Bio-Rad Laboratories). Supplemental Figure S4 displays total protein staining for each Western blot and the lanes crossed out with an “X” were extra samples that were not used in the final analysis for this manuscript. The blots were blocked in Tris-buffered saline (TBS) containing 0.1% Tween-20 (TBS-T) and 5% (*w*/*v*) bovine serum albumin (BSA) for 90 min at room temperature. The membranes were probed overnight at 4 °C with the primary antibody diluted in 5% (*w*/*v*) bovine serum albumin. All antibodies were purchased from commercial suppliers and were validated against known positive and negative expressing cell lines by Western analysis prior to use in experimental protocols. The source of the antibody along with their catalog numbers are reported in Table S2. After washing 5 times for 5 min each wash in TBS-T, the membranes were incubated with the anti-mouse or anti-rabbit secondary antibody (1:2000) for 90 min at room temperature. The blots were visualized using the ClarityWestern ECL Substrate (Bio-Rad Laboratories)

### 4.6. Immunohistochemical Staining

Intraperitoneal tumor tissue obtained from mice injected with non-treated and As^3+^-treated RT4 cells was used in this study. Serial sections were cut at 3–5 µm and immersed in preheated Target Retrieval Solution (citrate pH 6, Agilent) in a steamer for 20 min. The sections were allowed to cool to room temperature and immersed into TBS-T for 5 min. The primary antibodies used in this study along with their dilutions and catalogue numbers are listed in Table S2. The primary antibodies were localized using Dako peroxidase conjugated EnVision plus for rabbit or mouse primary antibodies at room temperature for 30 min. Liquid Diaminobenzidine (Dako) was used for visualization. Counter staining was performed using hematoxylin. Slides were rinsed in distilled water, dehydrated in graded ethanol, cleared in xylene, and cover slipped. Two pathologists judged the presence and degree of immune-reactivity in the specimens. The scale used was 0 to +3 with 0 indicating no staining, +1 staining of mild intensity, +2 staining of moderate intensity, and +3 staining of strong intensity.

### 4.7. Statistical Analysis

All experiments were performed in triplicate and the results are expressed as the mean ± SEM. Statistical analyses were performed using GraphPad Prism software (San Diego, CA, USA) version 8.2.1 using an unpaired *t*-test. Unless otherwise stated, the level of significance was *p* < 0.05.

## Figures and Tables

**Table 1:** Immunohistochemical analysis of As^3+^-exposed tumor heterotransplants.

	Parent		1 µM As^3+^	
Protein	INT	%	INT	%
PPARγ	3+	60	1+	20
FOXA1	1+	35	2+	60
GATA3	1+	50	2+	60
EGFR	1+	10	3+	80
TFAP2A	0	0	1+	60
P63	2+	30	3+	80
E-Cadherin	3+	90	3+	90
N-Cadherin	0	0	1+	10
ACTA2	1+	10	1+	30

INT: intensity of staining; %: % of cells staining for a marker; 3+: strong staining; 2+: moderate staining; 1+: weak staining.

## Data Availability

Not applicable.

## Supplementary Materials

The supporting information can be downloaded at: https://www.mdpi.com/article/10.3390/ijms232012313/s1.
