# Supplementary material for: Chronic Arsenic Exposure Upregulates the Expression of Basal Transcriptional Factors and Increases Invasiveness of the Non-Muscle Invasive Papillary Bladder Cancer Line RT4
Source: Int J Mol Sci. 2022 Oct 14;23(20):12313. doi: 10.3390/ijms232012313 (PMC9604142; doi:10.3390/ijms232012313)
Supplement: Supplementary file 1 [file ijms-23-12313-s001.zip › ijms-1960494-supplementary.pdf]

Supplementary Figure S1. Gene Expression in RT4 cells.

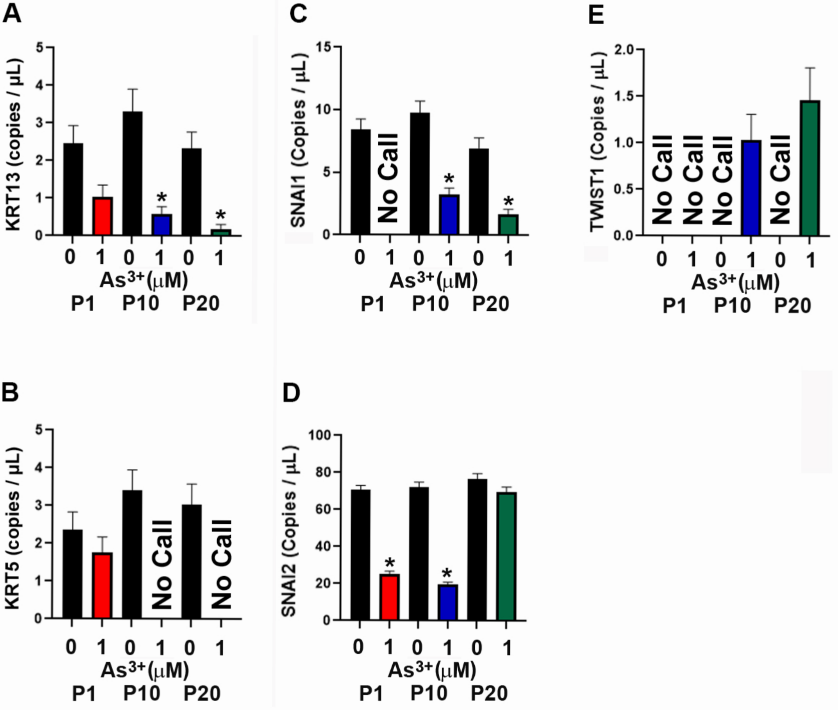

Supplementary Figure S2. Uncropped Western blots for Figures 2-4

Uncropped Western blots for Figure 2

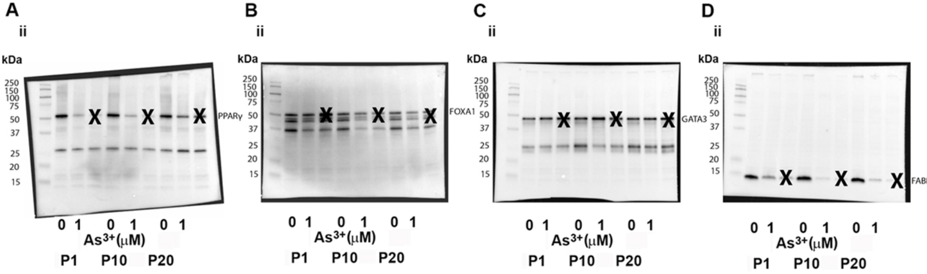

Uncropped Western blots for Figure 3

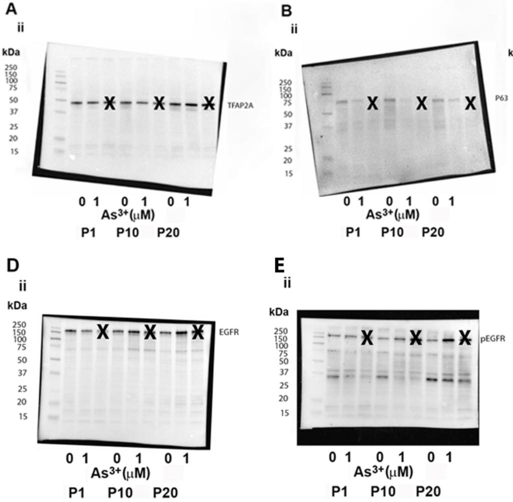

Uncropped Western blots for Figure 4

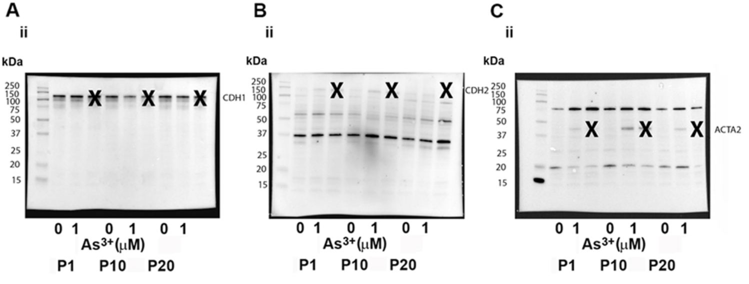

Supplementary Figure S3. Immunohistochemical analysis.

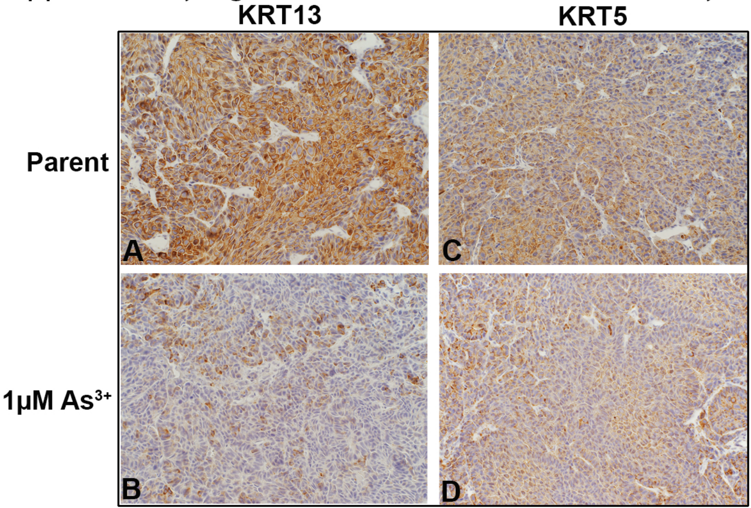

Supplementary Figure S4. Total protein loading for Western blot.

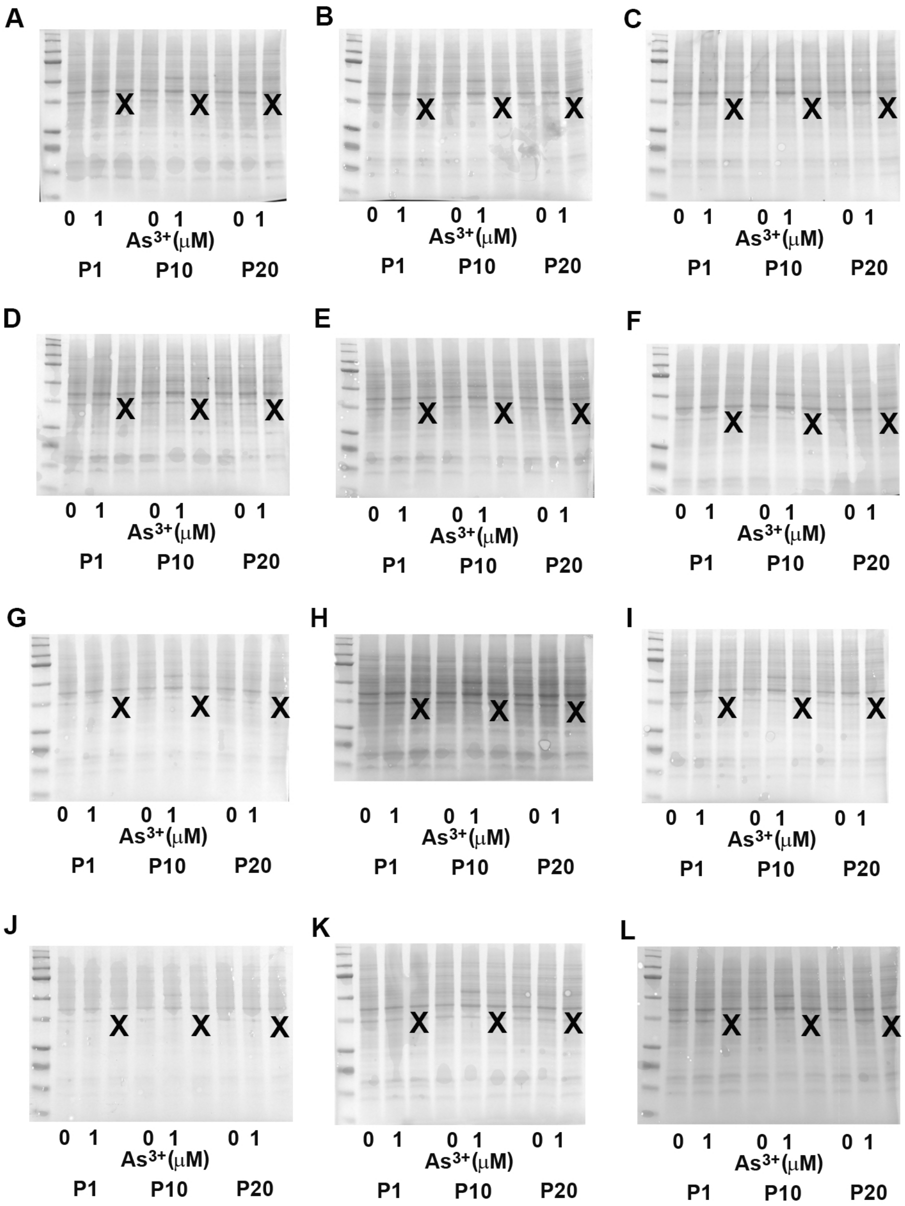

Legends for Supplementary Data

Figure S1. Gene expression in parent and As^{3+}-exposed RT4 cells. (A) KRT13 expression, (B) KRT5 expression, (C) SNAI1 expression, (D) SNAI2 expression, and (E) TWIST1 expression in parent and 1µM As^{3+}-exposed RT4 cells at passages 1, 10, and 20 (P1, P10, and P20). Droplet digital PCR (ddPCR) analysis was performed to verify gene expression. Gene expression is reported as copies per µL. Triplicate measurements of gene data was performed and are reported as mean ± SEM. Ordinary one-way ANOVA was performed followed by Dunnett's post-hoc test. A "no call" label indicates that expression was below limit of quantification of the instrument. Asterisks indicate significant differences from the passaged-matched parent ($p < 0.05$).

Figure S2. Uncropped Western blots for Figures 2-4. The total intensity for each protein band was quantified using Image Lab software (version 4.1, Bio-Rad Laboratories) and was normalized based on total protein loaded per lane. The lanes crossed out with an "X" were extra samples that were not used in the final analysis for this manuscript.

Figure S3. Immunohistochemical staining of keratins within the tumors formed from parent and As^{3+}-exposed RT4 cells. (A-C). Cytoplasmic expression of keratin 13 (KRT13) in tumors produced by the parent and As^{3+}-treated cells, the staining was strong in the parent and weaker in the tumors arising from As^{3+}treated cells. (D-F). Cytoplasmic staining of keratin 5 (KRT5), the tumors arising from the 1µM As^{3+}-treated cells appeared to have weaker staining compared to the tumors from parent cells. The brown color indicates the presence of the protein whereas the

blue/purple color indicates the nuclei that are stained with the counterstain hematoxylin. All images are at a magnification of 200X.

Figure S4. Total protein loading for Western blot analysis in parent and As^{3+}-exposed RT4 cells at passages 1, 10, and 20 (P1, P10, and P20). Total protein per lane for (A) PPARγ blot, (B) FOXA1 blot, (C) GATA3 blot, (D) FABP4 blot, (E) TFAP2A blot, (F) P63 blot, (G) TRIM29 blot, (H) phospho-EGFR blot, (I) EGFR blot, (J) CDH1 blot, (K) CDH2 blot, and (L) ACTA2 blot. The total intensities per lane (sum of all protein bands) were quantified using Image Lab software (version 4.1, Bio-Rad Laboratories) and used for normalization of target protein expression. The lanes crossed out with an "X" were extra samples that were not used in the final analysis for this manuscript.

Table S1

List of primers used in the study

Genes	Catalog No./unique Assay ID	Source
KRT5	qHsaCID0047798	BIO-RAD
KRT6A	qHsaCID0036985	BIO-RAD
KRT6B	qHsaCID0003069	BIO-RAD
KRT6C	qHsaCID0046712	BIO-RAD
KRT14	qHsaCID0047868	BIO-RAD
ACTA2	qHsaCID0013300	BIO-RAD
CDH1	qHsaCID0015365	BIO-RAD
CDH2	qHsaCID0015189	BIO-RAD
EGFR	qHsaCID0007564	BIO-RAD
FOXA1	qHsaCID0002547	BIO-RAD
GATA3	qHsaCID0017793	BIO-RAD
KRT13	qHsaCID0022608	BIO-RAD
FABP4	qHsaCID0036778	BIO-RAD
PPARG	qHsaCID0011718	BIO-RAD
TP63	qHsaCID0036332	BIO-RAD
TFAP2A	qHsaCED0044753	BIO-RAD
TRIM29	qHsaCED0005341	BIO-RAD

Table S2. Antibodies used in Western blot and immunohistochemistry analysis.

Antigen	Source	Cat. No	Western blot Dilution	Immunohistochemical Dilution
Keratin 5 (KRT5)	Invitrogen	PA5-29670	1:4,000	1:400
Keratin 6 (KRT6)	Santa Cruz Biotechnology	sc-514520	1:2000	1:400
Keratin 13 (KRT13)	Abcam	ab92551	1:100,000	1:200
Keratin 14 (KRT14)	Abcam	ab181595	1:20,000	--
EGFR	Cell Signaling Technology	4267	1:1000	1:50
pEGFR	Cell Signaling Technology	3777	1:1000	1:100
TFAP2A	Cell Signaling Technology	3215	1:1000	1:50
TRIM29	Abcam	ab244380	1:750	1:400
P63	BioLegend	687202	1:500	--
PPARG	Santa Cruz Biotechnology	sc-7273	1:250	--
FOXA1	Santa Cruz Biotechnology	sc-101058	1:250	1:50
GATA3	Cell Signaling Technology	5852	1:1000	1:250
FABP4	Santa Cruz Biotechnology	sc-271529	1:1000	1:50
E-Cadherin (CDH1)	Santa Cruz Biotechnology	sc-7870	1:1000	1:100
N-Cadherin (CDH2)	Invitrogen	PA5-19486	1:1000	1:100
ACTA2	Abcam	ab5694	1:1000	1:200
P63	Abcam	ab124762	--	1:1500
PPARG	Cell Signaling Technology	2435	--	1:400
Keratin 14 (KRT14)	Invitrogen	PA5-16722	--	1:400
